# A Delphi process to address medication appropriateness for older persons with multiple chronic conditions

**DOI:** 10.1186/s12877-016-0240-3

**Published:** 2016-03-15

**Authors:** Terri R. Fried, Kristina Niehoff, Jennifer Tjia, Nancy Redeker, Mary K. Goldstein

**Affiliations:** Clinical Epidemiology Research Center, VA Connecticut Healthcare System, 950 Campbell Avenue, West Haven, CT 06516 USA; Department of Medicine, Yale School of Medicine, 333 Cedar Street, New Haven, CT 06510 USA; Department of Quantitative Health Sciences, UMass Medical School, 368 Plantation Street, Worcester, MA 01605 USA; Yale School of Nursing, Yale University West Campus, P.O. Box 27399, West Haven, CT 06516 USA; Palo Alto Geriatrics Research Education and Clinical Center (GRECC), Veterans Affairs Palo Alto Health Care System, GRECC 182-B, 3801 Miranda Avenue, Palo Alto, CA 94304 USA; Center for Primary Care and Outcomes Research (PCOR), Stanford University, 117 Encina Commons, Stanford, CA 94305 USA

**Keywords:** Medications, Chronic conditions, Polypharmacy

## Abstract

**Background:**

Frameworks exist to evaluate the appropriateness of medication regimens for older patients with multiple medical conditions (MCCs). Less is known about how to translate the concepts of the frameworks into specific strategies to identify and remediate inappropriate regimens.

**Methods:**

Modified Delphi method involving iterative rounds of input from panel members. Panelists (*n* = 9) represented the disciplines of nursing, medicine and pharmacy. Included among the physicians were two geriatricians, one general internist, one family practitioner, one cardiologist and two nephrologists. They participated in 3 rounds of web-based anonymous surveys.

**Results:**

The panel reached consensus on a set of markers to identify problems with medication regimens, including patient/caregiver report of non-adherence, medication complexity, cognitive impairment, medications identified by expert opinion as inappropriate for older persons, excessively tight blood sugar and blood pressure control among persons with diabetes mellitus, patient/caregiver report of adverse medication effects or medications not achieving desired outcomes, and total number of medications. The panel also reached consensus on approaches to address these problems, including endorsement of strategies to discontinue medications with known benefit if necessary because of problems with feasibility or lack of alignment with patient goals.

**Conclusions:**

The results of the Delphi process provide the basis for an algorithm to improve medication regimens among older persons with MCCs. The algorithm will require assessment not only of medications and diagnoses but also cognition and social support, and it will support discontinuation of medications both when risks outweigh benefits and when regimens are not feasible or do not align with goals.

## Background

A growing body of evidence describes a number of problems with current patterns of prescribing for older persons with multiple chronic conditions (MCCs). These patients are underrepresented in randomized controlled trials providing the data underlying many disease management guidelines, and they may derive less benefit from and be at increased risk of harm from guideline-supported medications as compared to younger persons and those with lower chronic disease burden [1–3]. Older persons with MCCs take multiple medications and have complicated medication regimens, which increases the risk of drug-drug and drug-disease interactions [4] and can result in decreased medication adherence [5, 6]. Polypharmacy, variably defined according to the number of prescribed medications, has been associated in a large number of observational studies with an increased risk of a range of adverse events [7]. While it remains unclear whether this risk is due to the number of medications per se, there is preliminary evidence that medication deprescribing can improve outcomes [8, 9].

Current guidance for improving medication prescribing for older persons consists of either narrowly focused explicit medication review or broadly described implicit review based on general principles. Explicit criteria, such as the AGS Beers [10] and STOPP [11] criteria, provide specific recommendations for avoiding medications based on increased risks of adverse reactions in older persons and drug-drug/drug-disease interactions. The Medication Appropriateness Index (MAI) [12] provides additional criteria for implicit medication review, including indication, effectiveness, instructions, duplication and cost. Recognizing that additional factors affect the appropriateness of medication prescribing for the complex older patient, a number of investigators have expanded the basis for implicit medication review by proposing conceptual frameworks to evaluate appropriateness [3, 13–15] and to deprescribe medications [16, 17]. These frameworks include the concepts of evaluating time-to-benefit, patients’ preferences and goals and individually tailored benefits and harms or burdens. These frameworks provide a general description of the concepts without more specific strategies for applying these concepts to individual patients and regimens.

Similarly, interventions to improve medication regimens have either utilized explicit review to focus on a single medication/medication class or have utilized implicit review, requiring evaluations performed by pharmacists, physicians, and/or multidisciplinary teams.[18–20] The former type of intervention is easier to implement but is narrow in focus. The latter form of intervention, while comprehensive, is resource intensive and it is not known how individual practitioners apply the more general principles of appropriateness. Moving from general concepts or principles to more explicit strategies for identifying inappropriate medication regimens and deprescribing medications would help to fill this gap and serve as the basis for interventions that are both comprehensive and feasible. The purpose of this study was to develop a set of strategies or approaches for identifying and addressing problems with medication regimens for older adults with MCCs, focusing on the issue of deprescribing to reduce medication burden. In the absence of a strong evidence base, a Delphi panel was convened to obtain expert consensus.

## Methods

### Participants

Participants for the Delphi panel were selected to represent a broad range of expertise and perspectives as related to the care of the older person with MCCs. The nine panelists represented the disciplines of nursing, medicine and pharmacy. Physicians from a number of specialties were selected in order to ensure that any differences in perspectives among these specialties would be represented. Among the physicians, there were two geriatricians, one general internist, one family practitioner, one cardiologist and two nephrologists. Panelists were selected based on their record of scholarship regarding the management of older persons with MCCs and/or endorsement by a professional society as having expertise in this area. The first, second and last authors organized the work of the panel as part of the core research team but did not participate as members. The Human Subjects Committee of Yale University determined that the study qualified for exemption. Participants received an information sheet describing the study with assent indicated by completion of the Delphi survey.

### Study design

The panel used a modified Delphi technique [21, 22] to develop strategies for identifying problems with medication regimens for patients with MCCs and making changes in the regimens. The Delphi technique consists of iterative rounds of input from panel members, working anonymously, in which they are provided feedback from earlier rounds. The process included two modifications to the standard Delphi technique. The first was that the process began with a “time zero” panel meeting conducted by telephone. Although not generally used in the Delphi process, the time zero meeting has been used in the closely related RAND/UCLA appropriateness method [23]. One purpose of the meeting was to clarify the task to be addressed in the Delphi process, because of the complexity of the topic and the controversies regarding aging and its relationship to undertreatment versus overtreatment. The other purpose was to generate approaches to the identification and remediation of problems with medications among persons with MCCs. The second modification to the Delphi technique was that, rather than having the panel generate specific items, the core research team generated a candidate set of items for the panel to consider based on the results of the time zero meeting and literature review.

In preliminary work, the core team conducted two systematic reviews. The first of these reviews addressed the question of how the presence of comorbidity altered the benefits and harms of treatment for an index condition [24] and the second addressed the question of the adverse clinical outcomes associated with polypharmacy [7].

Prior to the time zero meeting, the panel received a document containing: 1) a statement of the problem; 2) the objectives for the Delphi process; 3) a summary of the systematic reviews; and 4) questions for the meeting. The panel was asked to consider three questions: 1) how do we know if there is a potential problem with a patient’s medication regimen? 2) how do we know if a regimen is consistent with a patient’s treatment goals? 3) what are approaches for reducing medications if there is agreement that the patient is taking too many?

Following the time zero meeting, the core team developed preliminary sets of items representing different strategies. These items were presented to panelists for their feedback through a website, with panelists providing their feedback anonymously. In the first round of the Delphi process, participants were asked to undertake one of two tasks for each of the items. For items that stated a fact, they were asked to rate their level of agreement with each item on a 5-point Likert scale: “Strongly Agree, Agree, Neutral, Disagree, Strongly Disagree,” and to provide comments to support their rating. For items seeking to establish a threshold for decision making, such as the level of hemoglobin A1C (HgbA1c) representing excessively tight control, they were asked to provide a number and to provide comments to support this number. Participants were invited to suggest changes to the language of the items and to provide additional items.

After the first round, responses were compiled to evaluate the level of consensus. Consensus was considered to be achieved for statements of fact if: a) all participants provided a rating of “agree” or “strongly agree;” and b) no participant recommended a change in wording. Consensus was considered to be achieved for threshold statements if all participants provided the same number. In the second round of the Delphi process, participants were presented with statements for which consensus was not achieved. Participants were also provided with their own and other panelists’ ratings/numbers and comments for these statements. They were given the task of re-rating/re-assigning a number based on the consideration of their own and others’ prior responses, as well as rating new items that were proposed during the prior round. The same procedure was followed for the third and final round. In each of the latter two rounds, eight of the nine participants provided ratings.

## Results

### Time zero meeting

In addition to providing content for the specific items described below, the discussion highlighted the limited data available to assess the risks and benefits of medication regimens and fit with patients’ goals. Panel members pointed out that there may be under-recognized effects of medications on outcomes that are important to patients, such as the beneficial effect of statins on walking performance and symptoms of claudication in patients with peripheral vascular disease [25, 26]. The panel also highlighted the problems of the exclusion of older persons with MCCs from clinical trials and the inappropriateness of extrapolating data from younger and healthier populations. The panel considered the fact that time to benefit is a very useful concept for improving decision making around cancer screening and diabetes management, but discussed the lack of evidence regarding the time to benefit for commonly used medications, such as antihypertensives and statins, for other chronic conditions. Finally, the panel felt that there was insufficient data to create strategies to tailor medication regimens to patients’ treatment goals. While they acknowledged that having patients prioritize their goals in terms of what was most important to them, they also discussed that it was unclear how to prescribe to best meet those goals.

### Development of the overall approach for identifying and addressing problems with medication regimens

**Fig. 1:**
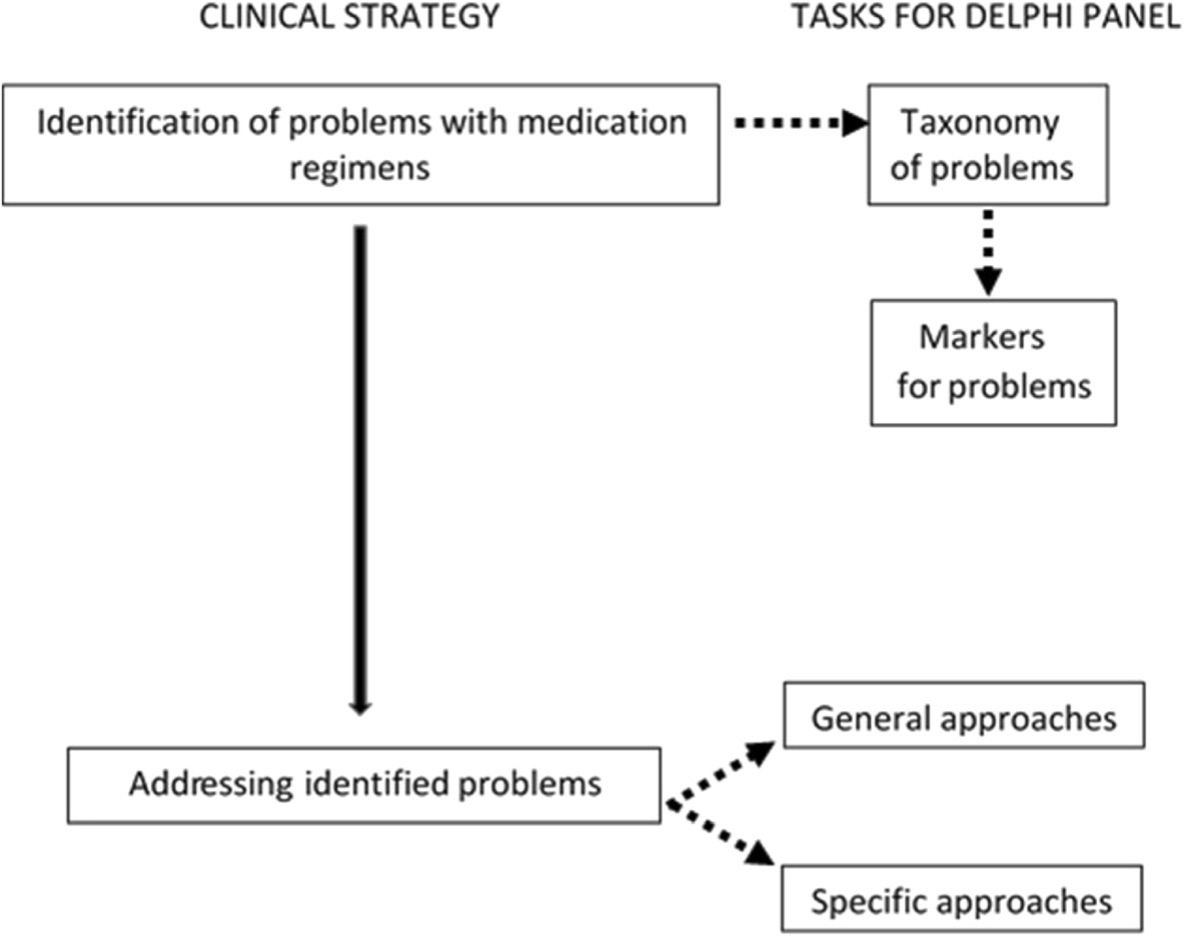
Clinical strategies to improve medication regimens among older persons and corresponding tasks for Delphi panel

The core team identified two steps necessary to organize the work of the Delphi panel: 1) development of strategies for identifying patients who have a problematic medication regimen, and 2) development of strategies for addressing the problem(s) with the regimen. The first step required developing both a potential taxonomy of problems with medication regimens and corresponding markers or indicators for each of these problems, since consensus could not be reached about how to identify a patient with a problem if there was not consensus about what the problems were. In order to avoid expanding the work of the Delphi panel beyond 3 rounds of ratings, the panel was asked to consider the taxonomy and corresponding markers simultaneously rather than sequentially. If consensus could not be reached on an element of the taxonomy, then the ratings regarding the markers were not further analyzed. Regarding the second step, the team recognized that the lack of available data would make it challenging to reach consensus regarding specific strategies for addressing problems, and therefore simultaneously developed a set of general strategies in addition to more specific ones (Fig. 1).

**Fig. 2:**
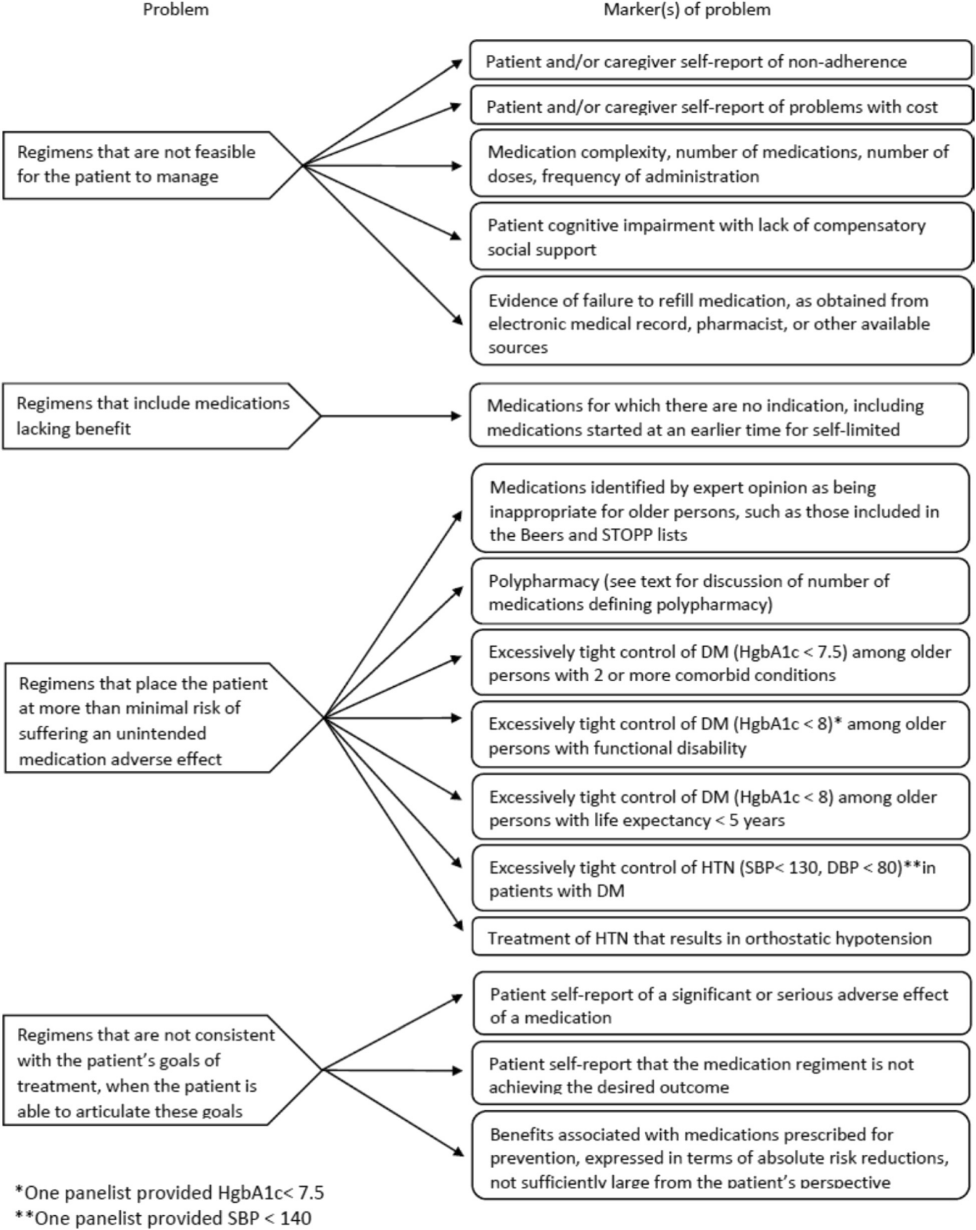
Problems with medications and their corresponding markers

### Delphi process: strategies for identifying problematic medication regimens

The Delphi panel reached consensus on a taxonomy of problems with medication regimens, including regimens that are not feasible for the patient to manage, regimens that contain medications with no benefit, regimens that place the patient at more than minimal risk of suffering an unintended adverse event, and regimens that are not consistent with patient goals. Details of the markers to identify each of these problems are provided in Fig. 2.

**Table 1:** Strategies for addressing problems with medication regimens

General approaches
• For the patient who is non-adherent because of issues of feasibility, it is advisable to reduce the total burden of medication in addition to providing increased support for taking medications, even including medications with known benefit.
• It is reasonable to undertake dose reduction or discontinuation of medications associated with both benefits and side effects if the patient views the side effects as more important than the benefits.
• It is reasonable to discontinue medications prescribed for primary/secondary disease prevention if the patient views the reduction of risk as insufficiently large.
• Clinicians and patients should have a discussion to prioritize patients’ goals for their medications and decide on a regimen that reflects this priority.
• Patients with polypharmacy even after steps are taken to discontinue medications should be informed of the risks associated with polypharmacy.
Specific approaches
• Medications on the Beers and STOPP lists should be considered for discontinuation, unless there is a compelling reason not to do so.
• Medications without an indication should be discontinued.
• A trial of dose reduction/discontinuation of medications with patient self-report of adverse effect should be undertaken, with re-evaluation to assess for improvement and possible rechallenge.
• Dose reduction/discontinuation of medications should be undertaken in patients with excessively tight control of DM.
• Dose reduction/discontinuation of medications should be undertaken in patients with excessively tight control of HTN.

While the panel agreed in Round 1 that polypharmacy was a marker for elevated risk, they did not reach consensus on the number of medications that constituted polypharmacy. In Round 1, the range of responses was from four or more medications to ten or more. In Round 2, the range narrowed from five or more to seven or more, but did not narrow any further in the final round. The panelists also agreed that excessively tight control of blood sugars and blood pressure in DM was a marker for increased risk, but the range of thresholds was similarly broad in Round 1. These ranges tightened considerably, so that consensus was either reached or almost reached by Round 3. Figure 2 includes items for which consensus for thresholds for HgbA1C and blood pressure was not fully achieved, but for which there was only one differing response that was in close range to the majority response.

### Delphi process: strategies to address medication problems

The general and specific strategies for which the panel reached consensus are presented in Table 1. In addition to discontinuation of medications with increased risk of harm, these strategies include the discontinuation of medications providing benefit depending upon the patient’s valuations of benefits and harms and the ability of the patient to manage the regimen. Consensus was not reached for several proposed strategies. These included the discontinuation of a number of vitamins and supplements that were identified by some Delphi panel members as having no evidence of benefit, such as multivitamins, gingko biloba and vitamin C. As described in the Time Zero meeting, the panel did not believe that there was sufficient evidence to create specific strategies based on the elicitation of patients’ treatment goals. During the panel process, there were concerns about the ability to convey risk information in a manner that the majority of patients would have an accurate understanding, and therefore could not reach agreement about strategies for discontinuing preventative medications based on patients’ valuations of the risk reduction they provide. In addition, while the panel agreed that there were risks associated with polypharmacy, there was no consensus achieved regarding the discontinuation of medications based on an evaluation of the cumulative risks and benefits of a regimen considered as a whole.

## Discussion

In the absence of a strong evidence base to guide the optimization of medication regimens for older persons with MCCs, a Delphi panel was convened to develop strategies for recognizing and addressing problems with medication regimens among this patient population. Before beginning the process of building consensus, the panel, participating in a time zero meeting, identified the lack of data as a major barrier to the work of the panel. Specifically, they pointed to the potential for under-recognized and under-studied benefits of medications on outcomes of importance to patients as well as the potential for harm and to the absence of data regarding time to benefit for many commonly used medications. They also identified a lack of data on how to tailor medication regimens to be consistent with patients’ goals. Nonetheless, the panel reached consensus on identifying a detailed specification of problems with medication regimens with markers delineating the clinical circumstances under which these occur. The work of the panel provides initial steps necessary for moving from general principles and concepts outlined in frameworks addressing the appropriateness of medications among older persons [3, 13–15] to an algorithm for medication review. The panel frequently had more difficulty reaching consensus about specific strategies for addressing these problems. Their endorsement of general strategies represents an important confirmation of principles that have been presented in the frameworks but have not yet been widely adopted in clinical practice. These include consensus about the need to consider discontinuation of medications even when they have known benefits, because of the presence of countervailing harms or burdens. The difficulties experienced by the panel in creating consensus-based detailed strategies for how to balance these considerations, particularly in the context of patients’ values, highlights the complexities of medication prescribing for older persons with MCCs.

The work of the panel made more strides in identifying strategies for how to *identify* a problematic medication regimen than for how to *address* those problems. Currently used criteria endorsed by the panel, including the MAI [12], STOPP [11], and AGS Beers [10] focus on the medication appropriateness as defined by the medications themselves and the patient’s medical diagnosis. The panel added criteria for identification of overtreatment of diabetes mellitus and of hypertension among persons with diabetes. This is in general agreement with a number of disease management guidelines [27–29]. The panel also further expanded the definition of appropriateness by endorsing additional strategies that evaluate the medication regimen according to patient factors reaching beyond the physiologic effects of the regimen. One dimension of this evaluation is an assessment of the feasibility of the regimen. Beyond the issue of cost, the panel identified the need to evaluate the complexity of the regimen, particularly in the context of the patient’s cognitive abilities and social support. By supporting an evaluation of the regimen in the context of the patient’s ability to adhere, the panel endorsed the notion that appropriateness is not determined solely by the effects of the medication. Following from this expanded notion of appropriateness, the panel endorsed a strategy of simplifying the regimen for patients who cannot compensate for the complexity of their regimens, even if this means discontinuing medications with clear benefits. Such an approach is a departure from the standard notion that medical appropriateness dictates the regimen, and interventions are aimed at the goal of having the patient adhere to that regimen [30]. The panel’s recommendations explicitly recognize the potential for irreconcilable conflicts between what may be medically indicated and what the older patient with cognitive impairment and limited support can reasonably manage, the only solution to which is to adjust the regimen.

A second dimension of the evaluation of appropriateness in the context of patient-related factors endorsed by the panel is the consideration of patients’ goals. This is not a new concept, as it has been included in a number of conceptual frameworks and/or guiding principles for managing medications among older persons [3, 13–15]. However, despite the widespread recognition of the need to tailor medications to patients’ preferences and goals, the panel was unable to come to agreement about specific approaches to do this beyond discontinuing medications with unacceptable adverse effects. The panel’s failure to develop additional specific strategies reflects the complexities of prescribing medications according to patients’ goals. First, there is little evidence of the effect of standard medical therapies on outcomes other than survival. While many older persons prioritize preservation of function over life prolongation [31], there are few data informing care planning to achieve this outcome. Second, while clinical experience may be able to guide clinicians whose patients desire a purely palliative approach to their care, this is not likely to be a common goal except for patients at the very end of life. In the absence of issues with feasibility, the burdens of medications may not be viewed as excessive, even by patients who are prescribed multiple medications. One study of patients’ views on the burden and benefits of medications suggests, not surprisingly, that the majority of older persons want a balance between the two and are willing to put up with multiple medications and some adverse effects for future benefits [32]. The ability to translate this desire for balance into a specific treatment plan is a multifaceted and complicated task the panel was unable to tackle.

The study has several limitations. While the Delphi panel consisted of clinicians representing a range of disciplines, it would have been even more comprehensive with the inclusion of additional subspecialties, such as endocrinology and rheumatology. Not all of the panelists provided ratings for all rounds, but each panelist participated in at least one round of ratings. With its focus on problems associated with polypharmacy and deprescribing, the Delphi process did not address the issue of failures to prescribe recommended medications, which is also highly prevalent among older persons [33].

## Conclusions

The results of the Delphi process used in this study provide specific strategies for identifying problems with medications, including recognition when medication regimens are not feasible, are associated with an excess risk of harm, and when they are not meeting the patient’s goals. Building on the work of individual investigators who have proposed frameworks for addressing medications among older persons, the strategies developed by the panel represent consensus opinion supported by a broad range of expertise. The strategies identified by the panel provide the first steps in implementing a clinical algorithm, based on a combination of explicit and implicit review, to address polypharmacy. The work of the panel suggests that this algorithm will require a comprehensive assessment of the patient’s medication regimen and health status; adherence with medications; patients’ reports of problems with medications and goals; and evaluation of the patient’s cognitive status and available social support. Additional work will be required to determine the best tools to use for this assessment. Based on this assessment, deprescribing can be done with varying levels of complexity and clinical judgment. The most straightforward involves discontinuation of medications without indications, those included in consensus-derived lists of potentially inappropriate medications, those leading to potential overtreatment of DM and HTN, and those for which adverse effects outweigh benefits. Many patients may still be left with a medication regimen that is not feasible and/or continues to confer the risks associated with polypharmacy. The fact that the panel struggled to reach consensus and find specific strategies for these patients suggests that further deprescribing will require complex clinical judgment in the context of what matters most to the patient.

## Availability of data and materials

The datasets supporting the conclusions of this article are available upon request to the corresponding author.
