# Erratum to: A Delphi process to address medication appropriateness for older persons with multiple chronic conditions

**DOI:** 10.1186/s12877-016-0300-8

**Published:** 2016-06-16

**Authors:** Terri R. Fried, Kristina Niehoff, Jennifer Tjia, Nancy Redeker, Mary K. Goldstein

**Affiliations:** Clinical Epidemiology Research Center, VA Connecticut Healthcare System, 950 Campbell Avenue, West Haven, CT 06516 USA; Department of Medicine, Yale School of Medicine, 333 Cedar Street, New Haven, CT 06510 USA; Department of Quantitative Health Sciences, UMass Medical School, 368 Plantation Street, Worcester, MA 01605 USA; Yale School of Nursing, Yale University West Campus, P.O. Box 27399, West Haven, CT 06516 USA; Palo Alto Geriatrics Research Education and Clinical Center (GRECC), Veterans Affairs Palo Alto Health Care System, GRECC 182-B, 3801 Miranda Avenue, Palo Alto, CA 94304 USA; Center for Primary Care and Outcomes Research (PCOR), Stanford University, 117 Encina Commons, Stanford, CA 94305 USA

## Erratum

After publication of this work [1], it was noted that there was an error within Fig. 2. Within this figure the word “problems” was omitted from the statement “Medications for which there are no indications, including medications stated at an earlier time for self-limited problems”. Figure 2 has been corrected in the original article and is also included correctly below.

**Fig. 2:**
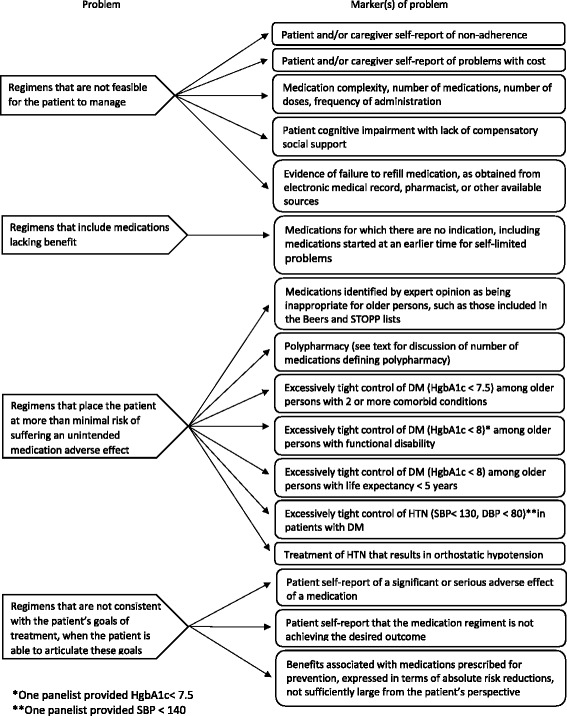
Problems with medications and their corresponding markers

## References

[1] Fried TR, Niehoff K, Tija J, Redeker N, Goldstein MK (2016). A Delphi process to address medication appropriateness for older persons with multiple chronic conditions. BMC Geriatrics..

